# Investigation of treatment delay in a complex healthcare process using physician insurance claims data: an application to symptomatic carotid artery stenosis

**DOI:** 10.1186/s12913-024-11860-w

**Published:** 2024-11-29

**Authors:** Stephen Christopher van Gaal, Arshia Alimohammadi, Mohammad Ehsanul Karim, Wei Zhang, Jason Sutherland

**Affiliations:** 1 Faculty of Medicine, University of British Columbia, 8151-2775 Laurel Street, Vancouver, BC V5Z1M9 Canada; 2 School of Population and Public Health, University of British Columbia, Vancouver, Canada; 3 Faculty of Pharmaceutical Sciences, University of British Columbia, Vancouver, Canada

**Keywords:** Routinely collected health data, Quality improvement, Process assessment, Health care, Data mining, Endarterectomy, Carotid, Amaurosis fugax

## Abstract

**Background:**

Delays in diagnostic and therapeutic processes are a potentially preventable cause of morbidity and mortality. Process improvement depends on accurate knowledge about as-is processes, historically collected from front-line workers and summarized in flowcharts. Such flowcharts can now be generated by process discovery algorithms supplied with chronological records from real-world cases. However, these algorithms may generate incomprehensible flowcharts when applied to complex unstructured processes, which are common in healthcare. The aim of this study is to evaluate methods for analysing data from real-world cases to determine causes of delay in complex healthcare processes.

**Methods:**

Physician insurance claims and hospital discharge data were obtained for patients undergoing carotid endarterectomy at a single tertiary hospital between 2008 and 2014. All patients were recently symptomatic with vision loss. A chronological record of physician visits and diagnostic tests (activities) was generated for each patient using claims data. Algorithmic process discovery was attempted using the Heuristic Miner. The effect of activity selection on treatment delay was investigated from two perspectives: activity-specific effects were measured using linear regression, and patterns of activity co-occurrence were identified using K means clustering.

**Results:**

Ninety patients were included, with a median symptom-to-surgery treatment time of 34 days. Every patient had a unique sequence of activities. The flowchart generated by the Heuristic Miner algorithm was uninterpretable. Linear regression models of waiting time revealed beneficial effects of emergency and neurology visits, and detrimental effects of carotid ultrasound and post-imaging follow-up visits to family physicians and ophthalmologists. K-means clustering identified two co-occurrence patterns: emergency visits, neurology visits and CT angiography were more common in a cluster of rapidly treated patients (median symptom to surgery time of 18 days), whereas family physician visits, carotid ultrasound imaging and post-imaging follow-up visits to eye specialists were more common in a cluster of patients with treatment delay (median time of 57 days).

**Conclusions:**

Routinely collected data provided a comprehensive account of events in the symptom-to-surgery process for carotid endarterectomy. Linear regression and K-means clustering can be used to analyze real-world data to understand causes of delay in complex healthcare processes.

**Supplementary Information:**

The online version contains supplementary material available at 10.1186/s12913-024-11860-w.

## Introduction

Timely access to healthcare services, therapies and technologies is a proven contributor to survival for cancer and cardiovascular disease, and an important determinant of patient experience [1–3]. Specific to symptomatic carotid stenosis, performing endarterectomy within two weeks of symptom onset reduces the absolute risk of stroke by 30%, compared with 18% for surgery performed after a delay of two to four weeks [4]. The Canadian Stroke Best Practice guidelines have strongly recommended a two-week target for endarterectomy since 2008 [5]. However, less than half of Canadian patients met this target in 2012, which is the latest year for which population-level data is available [6]. Recent single-center studies have reported that delayed treatment remains common, especially for patients presenting with vision loss rather than weakness or speech impairment [7]. The reasons for delay in this process are uncertain, and it is unclear how to approach their investigation.

The simplest tool for investigating delay in a healthcare process is the timeline – a chronological record of medical care for a single patient. Timelines can reveal certain problems such as repeated activities or excessive waiting times [8]. However, because timelines involve single patients, they cannot answer questions that depend on counterfactual ‘as compared to what’ reasoning, such as the optimal selection or sequencing of activities. Understanding these counterfactuals is important because physicians vary in their approach to patient care, particularly for use of diagnostic imaging and referrals to specialists [9]. Such understanding requires a method capable of summarizing and contrasting timelines for multiple cases. Most commonly, a flowchart is used.

Flowcharts precisely specify the selection and sequencing of activities. Gateways and branches identify decision points and illustrate consequences, providing the basis for comparing alternative workflows. The main drawback of flowchart models is their sensitivity to structuredness, or the number of unique process variants (see Supplementary Table 1). Structured processes with few variants yield simple flowcharts, whereas unstructured processes with many variants yield uninterpretable ‘spaghetti’ diagrams [10]. Structure can be imposed by reducing the number of variants, which may be done by filtering irrelevant activities, aggregating similar activities, or subsetting the population into groups with similar sequences [10–12]. However, these methods can obscure causes of variation in complicated processes. For example, it has been proposed that breast cancer treatment could be reduced to a 4-step process of medicines, visits, lab testing, and diagnostic imaging – a simplistic model that seems unlikely to help solve any problem related to breast cancer treatment [13]. 

In 2014, the Object Management Group released the Case Management Model and Notation (CMMN) as an alternative to the flowchart for modeling unstructured processes involving complicated or evolving scenarios, including clinical pathways for diagnosis and/or treatment [14]. In contrast to rule-based decision making typical of industrial processes, case management depends on judgment-based decision-making by knowledgeable front line workers (see Supplementary Fig. 1) [15]. CMMN models processes as sets of goal-based stages with associated activities; decisions on activity sequencing and selection are abstracted out of the models. This design allows complex processes to be represented in simple diagrams. However, the models cannot be directly used to compare the effects of alternative activity selection and/or sequencing choices.

Irrespective of format, the fidelity of models to real world processes depends on the accuracy and completeness of information. Historically, business analysts have manually created flowcharts based on interviews of front-line workers. However, finding and recruiting these individuals is not always practical, and their knowledge may be incomplete, especially for process that involve people working in different settings or organizations. Practitioners of ‘process mining’ posit that timestamped records of process activities, formally termed an event log, might be used in place of front line worker knowledge within a process analysis [10]. Since event log type data are routinely collected within physician claims and other cross-organization systems, process mining methods could allow investigation of processes that extend beyond single institutions [16]. 

Within the domain of process mining, specialized ‘process discovery’ algorithms may be used to generate flowchart-type processes using event log data. No process discovery algorithms have been specifically created to model non-flowchart processes and it is uncertain how they can be modeled from event log data. Frameworks, such as the Process Mining Project Methodology (PM^2^) have been developed to support analysts new to the domain [17]. Organizing frameworks such as PM^2^ might still be useful for guiding data-driven process analysis, even if there are no process discovery algorithms specifically designed for unstructured processes.

The aim of this study is to develop methods for analysing real world data from unstructured processes to identify process-related causes of treatment delay. In addressing this aim, we first assess whether algorithmic process discovery of an unstructured process yields an interpretable flowchart (RQ1). Next, we consider whether non-algorithmic methods based on CMMN models might help identify delays related to activity specific waiting times (RQ2.1) and to the selection of work activities (RQ2.2). As a case study, we use the symptom-to-surgery process for carotid stenosis presenting with vision loss. This is a suitable example for this aim because important delays are frequent, and the underlying process is complicated, typically involving multiple specialists working in different care settings. This is a novel and timely study as healthcare systems seek innovative approaches for reducing inefficiencies in their healthcare delivery models.

## Methods

### Study setting and cohort

The setting of this single-site study was the Vancouver General Hospital, a quaternary centre with a mixed catchment of local urban and rural/remote referral populations. The study cohort was created by querying the hospital’s discharge database for carotid revascularization codes (Canadian Classification of Interventions 1.JE.57, 1.JE.50, or 1.JE.87) between January 1, 2008 and December 31, 2016 [18]. We included patients who underwent endarterectomy for isolated retinal symptoms, and who had at least one claim within 60 days of recorded symptom onset. A publication from this cohort investigated models for the classification of symptomatic and asymptomatic carotid stenosis [19]. 

### Sample size

Sample size or power calculations were not performed for this exploratory study. Key methods used in this study, including process discovery algorithms and K-means clustering, lack a concept for statistical power.

### Framework

Process mining frameworks are intended to guide analysts toward the use of analytical best practices and away from common pitfalls [20]. All frameworks are organized as linear workflows, which universally include data extraction, data processing, and process mining stages. We followed the PM^2^ framework, which adds planning, knowledge-based process modeling, and process improvement stages [17]. We add a data quality step between processing and mining, as data quality problems are a common impediment to process mining but are not explicitly handled by PM^2^ [20, 21]. 

#### Planning

The main objective of this phase was to define the research question [17]. These are provided in the Introduction (RQ1-RQ2).

#### Knowledge-based process modeling

**Fig. 1:**
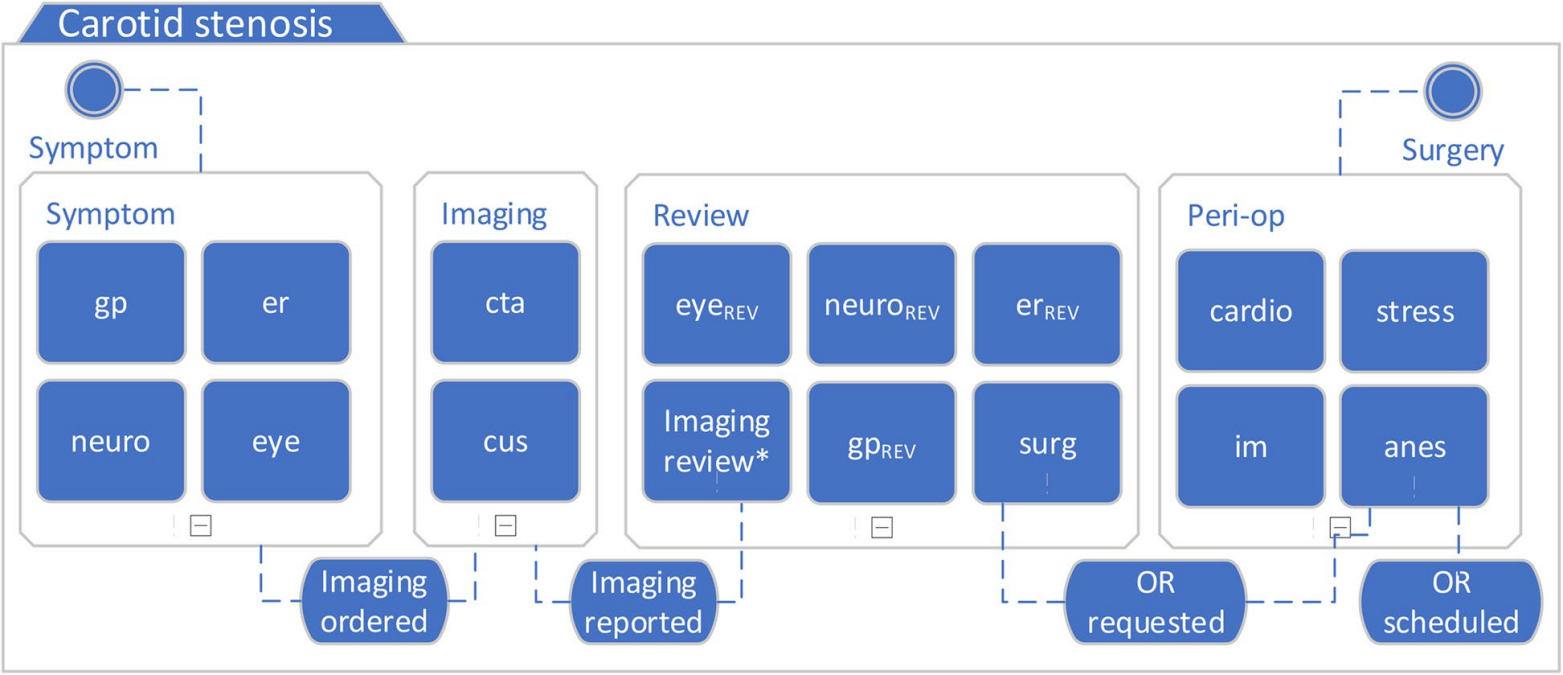
Case Management Model and Notation (CMMN) model of the symptom to surgery process for carotid endarterectomy. The process starts with patient symptom (top left) and ends when the surgery is complete (top right). There are four process stages, each with two to six potential activities. Exclamation points (!) signal required stages and/or activities. For example, patients must have at least one of CTA or CUS, but neither is specifically required. Some transitions may originate from any activity (e.g. imaging can be ordered by any physician), whereas OR (operating room) requested and OR scheduled must originate from surgery and anesthesia, respectively.  *Imaging review: imaging reports are returned to the ordering physician, who must decide on the next action. This might occur without an associated visit, so these activities might be structurally missing from physician claims data. See Supplementary Fig. 2

In this optional phase of PM^2^, knowledge and beliefs about the current process are collected from front line workers and process experts to guide data processing and analysis [17]. Using CMMN, a process model was created by a clinical stroke neurologist based at the site (SVG) and was reviewed with other site-based physicians. CMMN was used instead of a flowchart because event sequences were uncertain. Standard elements were specified including stages – phases of work, milestones – achievements and other states, and activities – work tasks (see Fig. 1) [22]. Activities were included if the physicians considered them relevant to the diagnosis and management of symptomatic carotid stenosis, such as carotid imaging. To reduce complexity, activities were excluded if they were considered unlikely to contribute to delay, either because they could be performed in parallel with carotid stenosis evaluation (e.g. if performed, a Holter monitor is unlikely to interrupt management of carotid stenosis) or because wait times are minimal (e.g. chest x-ray and laboratory tests). Activities were identified as mandatory if they were required for the carotid endarterectomy to proceed. Table 1 provides a list of stages, including goals and milestones, with available and mandatory activities. Referencing the insurer’s payment schedule, a crosswalk table of activities and associated physician service codes was created [23].

**Table 1:** Stage-based model for symptom-to-surgery process for carotid endarterectomy, including stage-specific goals, milestones, available activities, and mandatory activities

**Stage**	**Goal**	**Milestone**	**Available activities (abbreviation)**	**Mandatory activities and reason**
**Symptom **	Characterize symptom and identify potential causes	Imaging ordered	General practice (gp), emergency (er), optometry or ophthalmology (eye), neurology (neuro)	Stage must start with primary care (gp, er or optometry) – required for subsequent specialist visits
**Imaging**	Identify carotid stenosis	Imaging reported	Carotid CT angiogram (cta), carotid ultrasound (cus)	At least one of cta or cus – carotid imaging required for diagnosis and referral to surgeon
**Review**	Decide need for surgery	OR requested	Surgery (surg), neurology (neuro_REV_), optometry or ophthalmology (eye_REV_), general practice (gp_REV_), emergency (er_REV_)	Surgery (surg) – surgical consult necessary to trigger operating room (OR) request
**Peri-op**	Decide fitness for surgery	OR scheduled	Anesthesia (anes), cardiac stress test (stress), internal medicine (im), cardiology (cardio)	Anesthesia (anes) – required to schedule outpatient for surgery (not mandatory for unscheduled inpatient surgery)

#### Data extraction

In this stage, analytical data was compiled from primary sources (see Declarations) [17]. Medical charts were reviewed to confirm history of vision loss including symptom onset date and associated precision, specified within days, weeks, or months. Using each patient’s unique personal health number, medical chart data were linked with population-level datasets available through Population Data BC. These data included physician insurance claims (record of medical services including patient ID, insured service, ICD-9 diagnosis code, service date, claiming practitioner ID, referring practitioner ID and location type e.g. outpatient or inpatient); hospital discharges (admission and procedure date, and admission type as scheduled or unscheduled surgery); and demographics (age, sex, and in-metro residence). These data have near-universal population coverage due to the setting’s single-payer insurance system. Data were anonymized by replacing patient and practitioner ID numbers with study-specific ID numbers and by removing all quasi-identifiers except for dates. Results for subgroups with five or fewer participants are suppressed as a requirement of Population Data BC.

#### Data processing

##### Event log preparation

Using the fee schedule crosswalk, insured services were matched to knowledge-based process model activities (e.g. insured service 00407 Neurology office visit → neuro process activity). Variables from cross-walked claims were mapped to event log equivalents (patient ID → case ID; service date timestamp; insured service → process activity; claiming practitioner ID → resource; referring practitioner ID and ICD-9 diagnosis were included as additional fields).

##### Augmentation

Surgery and admission events were added based on hospital discharge data. Imaging review events were added for cases where there was no post-imaging visit to the referring physician for carotid imaging (see Supplementary Fig. 2).

##### Filtering and aggregation

Since patients might have visited their family physician or the emergency department for conditions unrelated to carotid stenosis, these visits were only included if they cited a relevant diagnosis code (as per Supplementary Table 2) or if they were linkable to a future referred activity by practitioner ID. For example, if a carotid ultrasound was referred by practitioner 1, we included the most recent, pre-ultrasound visit to practitioner 1 even if the diagnosis for this visit was irrelevant. Multiple instances of same-activity, same-practitioner, same-day events were reduced to a single instance.

##### Same day events

Process mining requires an unambiguously ordered event log. Because physician claims data is timestamped by date, the order of same-day activities is potentially ambiguous. To disambiguate the order of these events, combinations of same day activities were explored using UpSet plots (Supplementary Fig. 3) [24]. This identified recurring patterns in same-day activities, which could then be checked against known practice patterns. For example, the combination of same-day family physician and ophthalmologist visit was considered most likely to represent an initial visit to a family physician, followed by same-day referral to an ophthalmologist for vision loss. A standard order for same-day events was decided by a process expert based on this type of local practice knowledge (SVG). Ordering was implemented by adding an activity-specific time of day to each date (as per Supplementary Table 3).

##### Data quality

The comprehensiveness and validity of physician claims data as a record of process activities was investigated by checking for: (1) missing mandatory activities (as per Table 1), (2) referred activities listing a referring practitioner who did not have a preceding visit with the patient, and (3) surgical and anesthetic visits occurring before first carotid imaging, which is not allowed per our knowledge-based process model. We did not impute missing mandatory activities because they are plausibly structurally missing (not performed, rather than performed and not claimed). Patients with no claim for carotid imaging were excluded from linear regression and K-means analyses because the carotid imaging date is needed to correctly label symptom and review stage activities (e.g. neuro vs. neuro_REV_).

### Process analysis by research question

#### RQ1: Algorithmic process discovery

First, unique process sequences were counted as a measure of process structuredness. Next, process discovery was attempted using the HeuristicMineR implementation of the Heuristic Miner algorithm. This algorithm has commonly been used for mining healthcare processes and was the only algorithm available within the analytical environment [20]. It identifies causal connections between activities based on dependency, which is a measure of the consistency in the order of two events: if activity A reliably precedes activity B, and activity B never precedes activity A, then the dependency value for A→B is high, and A is considered a cause of B [25]. We enabled the recommended all-points-connected parameter [25]. To set the dependency threshold, a clinical stroke expert (SVG) assessed the plausibility of pairwise antecedent-consequent pairs from the dependency matrix. For example, emergency room visit to CT angiogram is a plausible sequence, whereas family physician to carotid endarterectomy is not (family physicians do not generally participate in the surgical decision-making). The threshold was set to the lowest, non-zero value observed for sequences considered plausible by the stroke expert (SVG). The algorithm generated waiting time estimates, activity and sequence counts, and petri-net diagrams, which resemble flowcharts. In a sensitivity analysis aimed at creating the simplest, most favorable conditions for process mining, we analysed only the symptom-stage activities of a homogenous group of cases identified using hierarchical clustering of event sequence Levenshtein distances [26, 27].

#### RQ2.1: Non-algorithmic measurement of activity specific waiting time

Calculating an activity waiting time requires request and service dates, but claims data only include the latter. We investigated two methods that determine request date based on the date of a preceding activity: directly-follows and referral matching. Directly-follows assumes that activities always occur in serial; each activity is requested by the directly preceding activity. This simple method underestimates waiting times for activities performed in parallel or with interruptions [28]. For example, a surgeon might request an anesthetic consult and stress test from the same visit (parallel), or a patient might return to a family physician for worsening symptoms while waiting for a previously requested consult (interruption). The availability of the referring practitioner ID field in our dataset allowed us to link events to starting points based on matching practitioner ID. This referral-match method can account for parallel activities but not interruptions, and missing results due to unmatched practitioner ID could be problematic. Waiting times calculated using each method were compared using pairwise t-tests. The proportion of unsuccessful referral matches was calculated for each activity. Origin-specific waiting times (e.g. gp to cus vs. neuro to cus) were calculated using referral-matched data and compared using non-parametric tests.

#### RQ2.2: Non-algorithmic measurement of the effect of activity selection on waiting time

##### RQ2.2 – Analytic goal: identify high-impact decisions

In an unstructured process, front line workers make decisions about activity selection and sequencing in real-time, based on their expertise and knowledge about each specific case. Some decisions may impact the selection, sequencing or prioritization of later activities, resulting in a disproportionate impact on cumulative waiting time. For example, a family physician’s decision to refer a patient to neurology will often culminate in urgent CT angiography and same-day admission for unscheduled surgery, whereas this type of priority evaluation is less likely if the patient is initially referred to an ophthalmologist. Unfortunately, modeling such decisions within unstructured processes is difficult due to the combinatorial explosion that results from stacking multiple decisions. A simpler alternative is to consider the relationship between activity selection and waiting time. We investigated this from two perspectives: individual activities and activity co-occurrence.

##### RQ2.2 – Analytic outcome: stage-forward waiting time

**Fig. 2:**
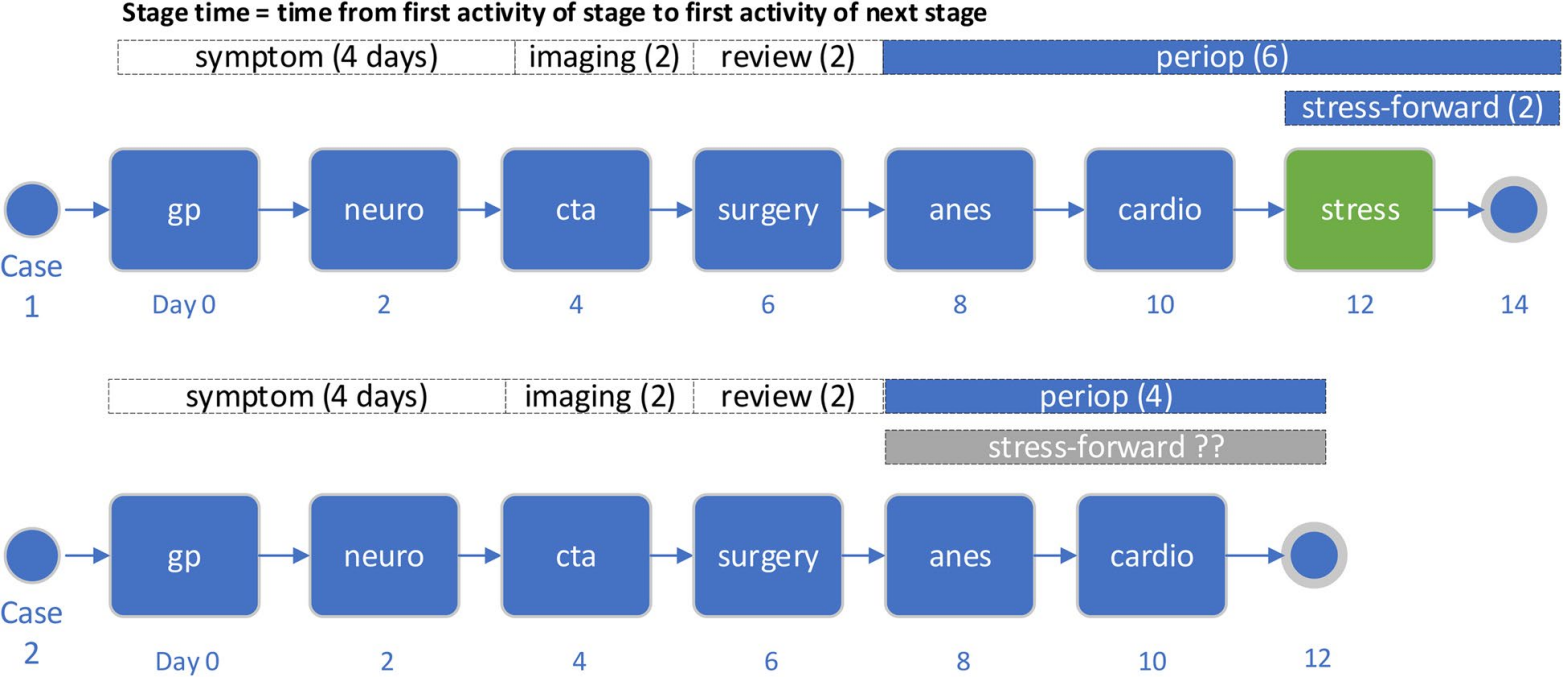
Illustration of stage-forward time applied to the stress test activity. If a stress test is to be performed it will be one of the last activities in the process (green box). To understand the impact of stress tests on waiting time, we need to be able to compare patients with stress tests against patients without one (the counterfactual). The simplest comparison is total time (14 days vs. 12 days), but this is confounded by delay accumulated in the preceding symptom, imaging, and review stages. Time from stress test to surgery is unconfounded (stress-forward time, 2 days), but the counterfactual time for the patient without the stress test is uncertain. Stage-forward waiting time (6 days vs. 4 days) is less confounded than total waiting time and is calculable for all patients

In examining the impact of individuals’ activities on carotid stenosis waiting time, one option is to compare total waiting time for patients with and without an activity. However, this approach cannot distinguish waiting time that accumulates after the activity, which is potentially attributable to the activity in question, from waiting time that accumulated before the activity, which is not attributable and is a potential confound. Because the intent of this analysis is to identify activities that lead to particularly timely or delayed care, we want to minimize noise from upstream (before activity) delays. This is particularly important for activities that occur late in the process, such as cardiac stress tests. Unfortunately, time from the activity to the end of the case cannot be used, since this cannot be measured for cases without the activity. As an alternative, we use stage-forward times when evaluating activity-specific effects (Fig. 2).

##### RQ2.2 – Analysis of single activity effects

Stage-forward waiting times were compared for patients with and without individual activities using the Wilcoxon rank-sum test. Linear regression was used to test for multivariate association between stage-forward time and stage-based activities, including sex, age, in metro residence (yes/no), and year of endarterectomy as additional covariates. Unscheduled admission allows review- and perioperative-stage activities to be rapidly performed, so it was included as a variable in models for these stages. Two sets of sensitivity models were created: (1) adding pre-stage waiting time to account for possible de-prioritization of cases already delayed, and (2) removing the unscheduled admission variable. Confidence intervals for coefficients were calculated using 1,000 nonparametric bootstraps.

##### RQ2.2 – Analysis of multi-activity effects

K-means clustering was used to identify co-occurrence patterns in activity selection. For each patient, each activity was counted as observed or not observed, yielding a binary matrix of n patients x m activities. The optimal number of clusters was determined by gap statistic using 1,000 bootstrap samples [29]. The entire population was clustered. Patient characteristics and total waiting time were compared for clusters of at least *n* = 6. Jaccard values of cluster stability were determined using 1,000 bootstrap samples from the clustered population, with values < 0.5 were considered unstable [30]. Between-cluster differences in activity frequency were compared for the two largest clusters, using a new set of 1,000 bootstrap samples from the clustered population.

## Results

Characteristics of ninety included patients undergoing carotid endarterectomy for retinal ischemic symptoms between 2008 and 2016 are presented in Table 2. The participant flow diagram is provided in Supplementary Fig. 4.

**Table 2:** Characteristics of included patients. Measurement data are presented as median (Q1 – Q3) and counts as N (%). Many symptom onset dates were recorded with weeks or months precision, likely explaining the discrepancy between symptom onset to surgery vs. first relevant claim to surgery, particularly at the higher bounds (Q1 unaffected)

**Characteristic**	**N = 90**
Age	75 (67, 80)
Female sex	27 (30%)
In metro residence	77 (86%)
Charted symptom onset date to surgery, days	34 (16, 71)
Precision recorded in days	50 (56%)
First relevant claim date to surgery, days	42 (17, 78)
First carotid imaging ordered by family physician	28 (31 %)
Surgery within 14 days of chart symptom onset date	19 (21%)
Surgery as unscheduled procedure	33 (37%)

An initiating activity other than primary care was identified for 15 patients, most commonly a visit to an ophthalmologist previously visited by the patient (*n* = 7). Fewer than six patients were missing carotid imaging or the surgical or anesthetic visits. Fewer than six patients had an anesthesia or surgical visit before imaging.

### RQ1: Algorithmic process discovery

Every patient had a unique sequence of events. Considering only the four symptom-stage activities of a ‘primary care’ cluster of 53 similar patients, there were still 35 unique sequences, with only one sequence observed in more than five patients (sequence {gp}, 7 patients).

Based on the review of antecedent-consequent activity pairs, the dependency threshold was set at 0.2 (Supplementary Fig. 5). Frequency maps yielded plausible counts of activities, but sequence counts were incorrect due to double counting (Supplementary Fig. 6). Waiting time maps generated errors unless constructed from subgroups of fewer than 30 patients; a map of symptom activities for 25 primary care cluster patients was unintelligible (Supplementary Fig. 7). Flowchart-type petri-nets were incomprehensible ‘spaghetti’ diagrams; the simplest model of four symptom-stage activities in the primary care cluster included 71 connecting arrows (Supplementary Fig. 8).

### RQ2.1: Non-algorithmic measurement of activity specific waiting time

**Fig. 3:**
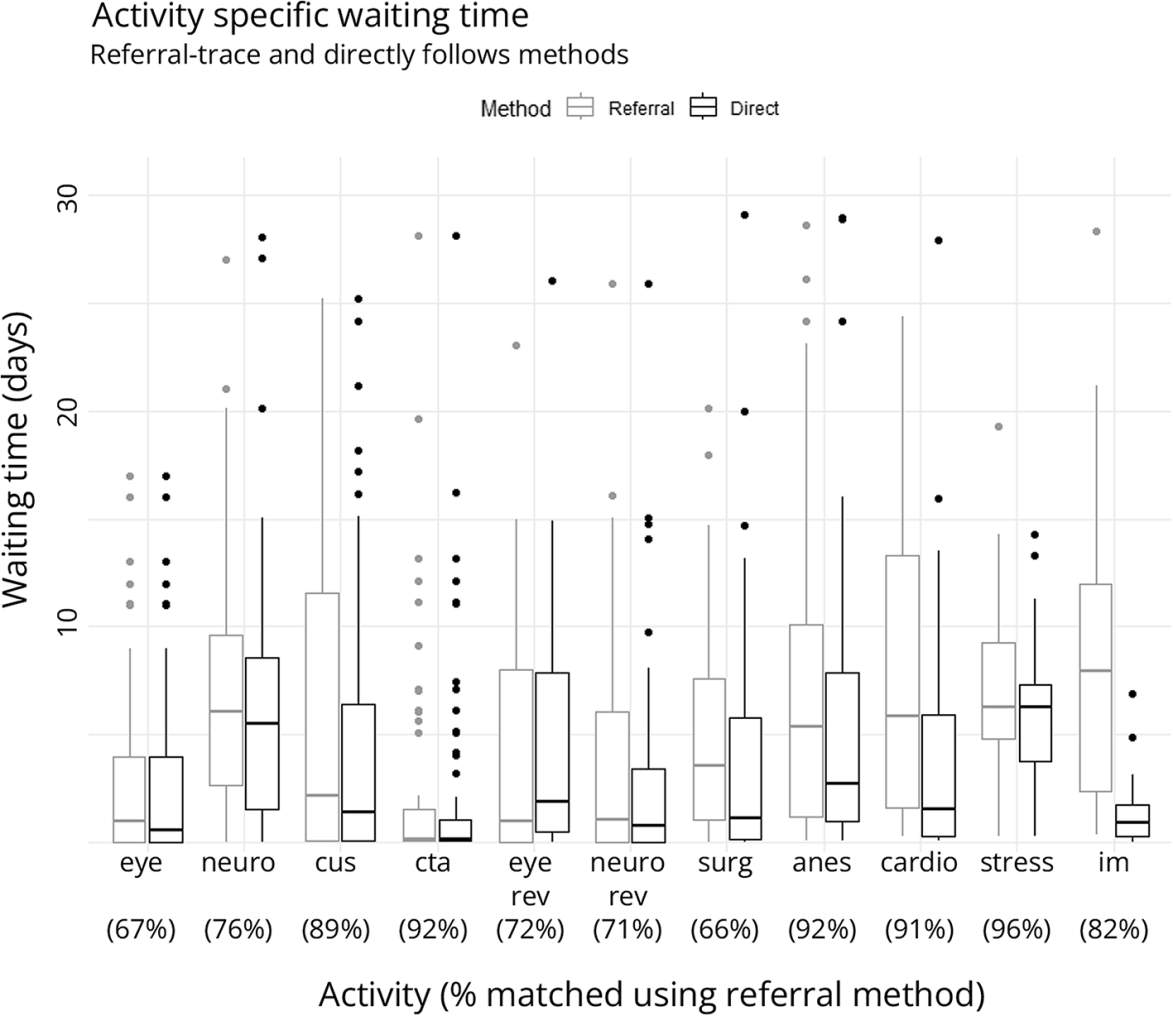
Activity specific waiting times by referral-trace and directly-follows methods. Percentage of successful referral-based matches at bottom of box plots

Compared to the referral-based method, waiting times calculated by the directly-follows method were significantly shorter, except for ophthalmology/optometry visits and carotid ultrasound (Fig. 3, Supplementary Table 4). Referral matches were consistently identified for activities in imaging and perioperative stages, but less consistently for symptom and review stage activities. Waiting time was shorter for CT angiography compared to carotid ultrasound. Waiting times were generally longer if referred by general practitioners or eye specialists as compared to emergency physicians, neurologists, and surgeons (Supplementary Table 5, Supplementary Fig. 9). This discrepancy was especially pronounced for carotid ultrasound. Notably, only eight carotid imaging investigations were ordered by ophthalmologists, all of which were ultrasounds.

### RQ2.2: Non-algorithmic measurement of the effect of activity selection on waiting time

**Fig. 4:**
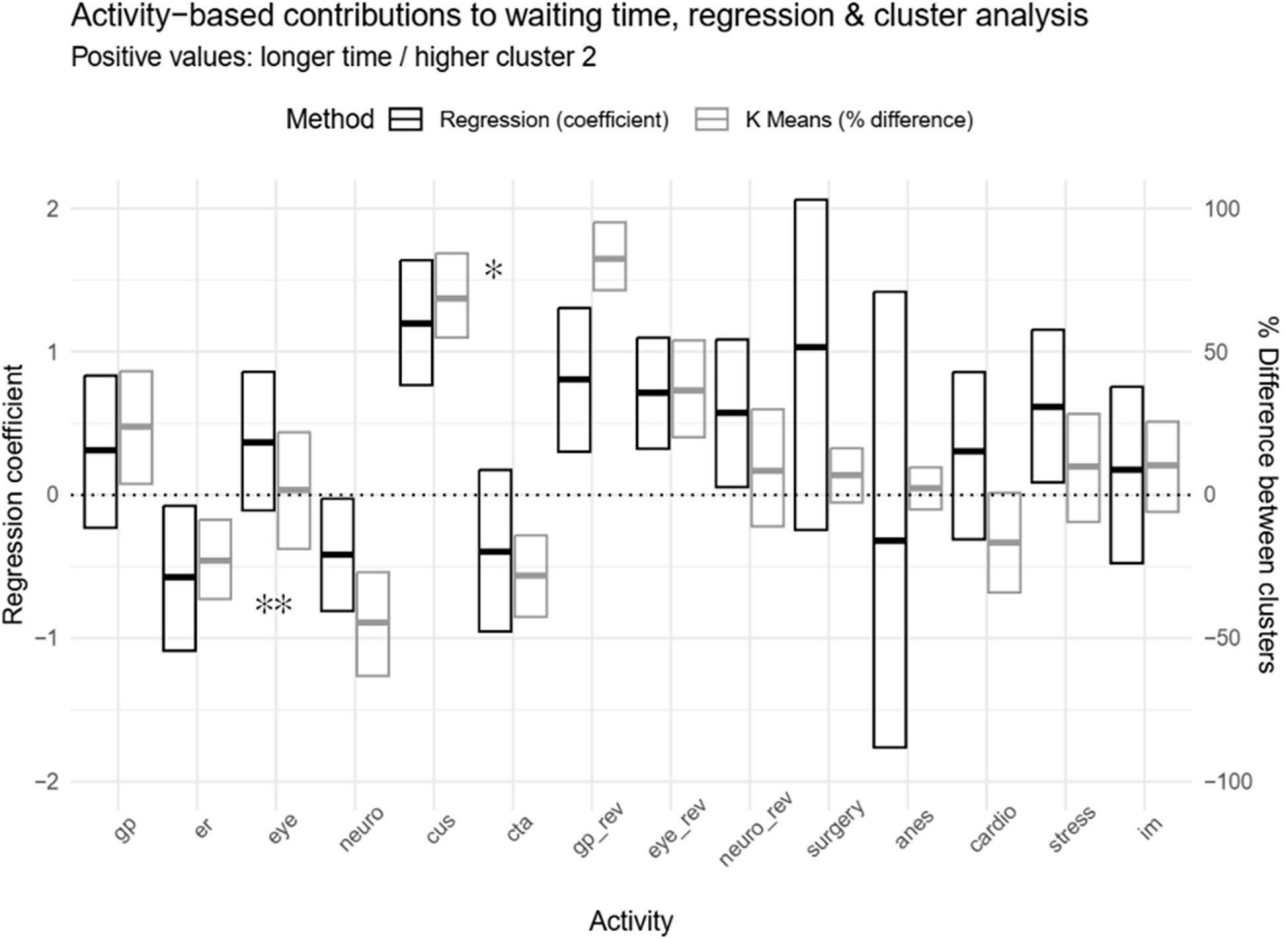
Linear regression and K-means clustering analyses of activity occurrence. Activity-specific linear regression coefficients for stage-based forward waiting time appear in black (positive values associated with longer time, left y-axis). Between cluster differences in activity occurrence are illustrated in grey (positive values more frequent in Cluster 2, right y-axis). Boxes denote bootstrapped 95% confidence intervals based on 1,000 replicates.  * cus, gp_rev and eye_rev were associated with longer stage-forward time and were more frequent in Cluster 2.  ** er and neuro were associated with shorter stage-forward time and were more frequent in Cluster 1.

Activity presence, quartiles of activity counts and univariate association with stage-forward time are presented in Table 3. Multivariate linear regression models were variably predictive of stage-forward time (95% bootstrapped CI for R^2^: symptom – 0.08–0.34; imaging – 0.17–0.47; review – 0.49–0.73; periop – 0.31–0.58). Confidence intervals for activity regression coefficients are graphically summarized in Fig. 4. Coefficients were similar between the two sets of stage-based sensitivity models, with modest differences in model R^2^ up to 0.12.

**Table 3:** Activity-based analysis of symptom-to-surgery process and association with stage-forward waiting time

**Stage**	**Activity name (abbreviation)**	**1+ activity N (%)**	**# activities Median (Q1 - Q3; max)**	**Stage-forward waiting time Median (Q1 – Q3)**		
				Activity present	Activity absent	*P*-value*
**Symptom**	General practice (gp)	58 (67%)	1 (0 – 1; 9)	63 (34 – 118)	25 (17 – 61)	0.011
	ER (er)	14 (16%)	0 (0 – 0; 1)	24 (16 – 39)	61 (26 – 120)	0.005
	Optometry or ophthalmology (eye)	56 (64%)	1 (0 – 2; 7)	55 (29 – 106)	35 (14 – 100)	0.2
	Neurology (neuro)	49 (56%)	1 (0 – 1; 2)	38 (18 – 67)	67 (37 – 113)	0.079
**Imaging**	Carotid ultrasound (cus)	52 (60%)	1 (0 – 1; 1)	49 (27 – 105)	12 (6 – 26)	<0.001
	Carotid CT angiogram (cta)	78 (90%)	1 (1 – 1; 3)	27 (11 – 49)	61 (51 – 106)	0.004
**Review**	General practice (gp_REV_)	39 (45%)	0 (0 – 1; 4)	43 (29 – 93)	12 (5 – 20)	<0.001
	Optometry or ophthalmology (eye_REV_)	25 (29%)	0 (0 – 1; 7)	43 (30 – 101)	17 (5 – 35)	<0.001
	Neurology (neuro_REV_)	53 (61%)	1 (0 – 1; 6)	24 (11 – 47)	25 (4 – 45)	0.3
	Surgery (surg)	82 (94%)	1 (1 – 2; 3)	N/A (at least one cell N ≤ 5)		
	Unscheduled admission	30 (33%)	N/A	7 (12 – 37)	20 (4 – 16)	<0.001
**Periop**	Anesthesia (anes)	84 (96%)	1 (1 – 1; 2)	N/A (at least one cell N ≤ 5)		
	Cardiologist (cardio)	21 (24%)	0 (0 – 0; 2)	15 (5 – 36)	16 (6 – 26)	0.4
	Cardiac stress test (stress)	24 (28%)	0 (0 – 1; 1)	36 (18 – 71)	9 (5 – 21)	<0.001
	Internal medicine (im)	17 (20%)	0 (0 – 0; 2)	20 (15 – 65)	14 (5 – 29)	0.057

* Wilcoxon rank-sum test

Analyses identified two clusters based on the bootstrapped gap statistic. Both clusters were stable (both Jaccard = 0.81). Cluster 1 patients had shorter symptom-to-surgery time, and were younger, more likely to have had unscheduled surgery, and less likely to have had imaging ordered by a family physician (Supplementary Table 6). Differences in activity occurrence between clusters are graphed alongside regression coefficients in Fig. 4.

## Discussion

Using the symptom-to-surgery process for carotid endarterectomy as a case study, our overarching goal was to identify a set of methods useful to the investigation of delay in access to care in an unstructured process. We believe that to be useful in the context of this goal, an analysis must help elaborate and explain recommendations about the selection, sequencing and/or resourcing of work in relation to delay.

As concrete context, consider two key findings from a recent survey of neurologists and ophthalmologists regarding the management of recent vascular-type vision loss: (1) 72% of neurologists would arrange immediate hospital-based investigation vs. 21% of ophthalmologists and (2) 1% of neurologists would delegate workup to another physician vs. 41% of ophthalmologists [9]. Based on these survey data, one might infer that an initial decision to refer to neurology instead of ophthalmology will likely result in faster diagnosis and treatment, and that the reason for this advantage relates to different practice patterns for emergency department referral and task delegation. We believe that a useful analysis will reveal these types of decisions and practice patterns from real world data without requiring a priori hypotheses from surveys or other data sources.

Our first approach was to try algorithmic process discovery. We used the Heuristic Miner, which is commonly used for the analysis of healthcare process [20]. Unfortunately, the flowchart-type diagrams generated by this algorithm were complicated and no conclusions could be drawn about the potential causes of delay. Flowchart complexity depends on the number of unique sequences, and every patient’s sequence was unique [25]. The number of unique sequences could have been reduced by further aggregating activities, such as visits to all specialists [13]. However, this approach would make the detection of speciality-specific differences difficult, including any difference between neurologists and ophthalmologists. The number of unique sequences could also have been reduced by limiting scope and subsetting similar cases [10]. However, this approach did not work for our data – flowcharts remained complicated even after scoping the process to four symptom-stage activities and subsetting by cluster similarity. As the sensitivity to sequence number is a property of flowcharts in general, and all common process discovery algorithms generate flowchart-type outputs, we are uncertain whether algorithmic process discovery will prove useful to the understanding of delay in unstructured processes.

Compared to flowchart models, CMMN models are less sensitive to sequence number. However, as CMMN models lack semantics for decisions and sequences, they cannot be directly used to understand delay in a process. Although CMMN modeling is not sufficient for analysis, it was essential to our method in four respects. First, by organizing work into a series of goal-based stages, CMMN modelling provided a rational basis for activity selection and aggregation. Second, activities could be classified according to goal: our distinction between neurology visits in the symptom evaluation and post-imaging review stages is based on the CMMN model. Third, CMMN models embed logic, such as potentially substitutable activities, that is necessary for understanding counterfactuals and decisions. Fourth, CMMN modeling allowed logical organization of our statistical analysis, such as the display of model outputs using a standard timeline. However, it is unclear which statistical methods might be useful to the analysis of a CMMN-modeled process. We considered methods related to two perspectives: activity-specific waiting time and activity selection.

For the activity waiting time analysis, our goal was to identify activities with unusually long waiting times. We compared two methods: directly-follows, which is commonly used in process analysis, and referral-match, which might be novel [28]. Both methods yielded similar estimates of waiting time, except for perioperative activities, where parallel processing of activities might have been more common. We think the estimated waiting times could be useful in two respects: (1) they could inform a choice between substitutable activities, such as ultrasound or CT-based carotid imaging, and (2) they could inform decisions about resource allocation by identifying activities with problematic wait times. However, activity waiting times do not identify complex causes of delay, such as delays related to the practice patterns of different specialists.

An unexpected benefit of the referral-match method is explicit tracing of provenance. This allowed waiting times for diagnostic tests and consults to be stratified by referral specialty, which revealed a substantial advantage for tests and consults referred by specialists or surgeons. This finding is not readily corroborated in the literature and warrants further study, ideally based on the analysis of source documents including requisitions, referral forms, and triage decisions. Provenance is likely to be useful to the identification of practice patterns. For example, we demonstrate that carotid imaging is rarely ordered by ophthalmologists, even though they are frequently involved at the symptom evaluation stage. The importance of activity provenance will re-emerge in the subsequent discussion of activity selection.

For the activity selection analysis, our goal was to identify specific decisions that have large positive or negative impacts on waiting time. For reasons outlined in the Methods, we believe that this is a difficult analytical task. We believed that linear regression and K-means clustering might offer complementary perspectives. Linear regression estimates the effect of individual activities on waiting time. Although every activity has a non-zero waiting time, activities can have an overall negative impact on total waiting time if they are associated with efficient workflows. K-means clustering identifies groups of cases that share similar attributes. In this case, we cluster based on presence or absence of each activity, revealing whole-workflow patterns in activity co-occurrence. Because symptom-to-surgery times were significantly different between clusters, it was reasonable to relate these whole workflow patterns to delay.

When interpreted in the context of the CMMN process model, these analyses are likely useful to understanding delay. With respect to the survey example, the CMMN model identifies a decision between the substitutable neurology and ophthalmology activities; selecting the neurology activity is straightforward given its negative regression coefficient and over-representation in the fast-cluster group. However, insights from these methods may be limited, especially where provenance is important. For example, the CMMN model identifies carotid imaging with ultrasound and CT angiography as substitutable activities. Thus, upon finding that ultrasound is associated with delay, we might think that imaging with CT might be faster. However, if this delay is related to the ordering of imaging by a family physician, then the fastest course of action might be to defer carotid imaging to a specialist. Informed by the CMMN model, the activity waiting time and activity selection perspectives would likely reveal the types of decisions and practices discussed in the survey and are therefore likely to be useful for analysis of unstructured process.

This study has important limitations. First, this is a single center study involving a single disease condition, so our methods may not generalize to other types of unstructured process. Second, due to restrictions of our analytical environment, we were unable to use ProM or pm4py software, which implement additional process discovery algorithms including the fuzzy and inductive miners [10]. However, because these algorithms still depend on flowcharts, we do not think that other algorithms would have proven more insight into the causes of delay. Third, we did not have access to medical records of community physicians, precluding validation of our waiting time estimates. We specifically cannot assess the frequency of interruptions or their impact on waiting time estimates. Groups with access to administrative data and community medical records are well positioned to corroborate these findings, perhaps by comparing known referral and service dates to dates inferred from claims data [31]. Finally, the design of this study likely excludes most patients with carotid stenosis who have an ischemic stroke while waiting for surgery, as these strokes frequently lead to disqualifying morbidity. Therefore, our knowledge of the most ineffective workflows remains limited.

There remains unmet need for methods capable of applying data to the management and improvement of unstructured processes that are common in health care. Although many groups have used process discovery in the healthcare context, few have described the use of process mining to identify and resolve a process-related cause of a specific problem, such as treatment delay [20, 32]. For example, Stefanini et al. used the Heuristic and Inductive Miners to generate a process map of the services provided to lung cancer patients; they reported that the resulting flowchart was considered useful by hospital staff, but the scope of the process was unclear and they did not report on the application to any specific process problem such as cost, quality or delay [11]. Similarly, Mans et al. were able to use the Heuristic Miner to generate a flowchart of a gynecology process, but without clear articulation of clear process scope or problem to be solved [33]. Additional literature is presented in Supplementary Table 7. We believe that clinical involvement in process analysis is indispensable; concepts such as clinical goals and activity relevance are essential to the design and interpretation of process models.

## Conclusion

Flowchart-based process discovery algorithms may be ill suited to the investigation of delay in unstructured healthcare processes. Case management modeling may be used in conjunction with common statistical techniques to understand process delay related to activity waiting times and activity selection.

## Supplementary Information

Supplementary Material 1. [34–37]

## Data Availability

The data that support the findings of this study are available from Population Data BC, but restrictions apply to the availability of these data, which were used under license for the current study, and so are not publicly available. Data are however available from the authors upon reasonable request and with permission of Population Data BC. Per Population Data BC: Access to data provided by the Data Stewards is subject to approval but can be requested for research projects through the Data Stewards or their designated service providers. The following data sets were used in this study: Medical Services Plan Payment Information File, Discharge Abstract Database and Consolidation File. Further information regarding these data sets can be found by visiting the PopData project webpage at https://my.popdata.bc.ca/project_listings/19-073/collection_approval_dates. All inferences, opinions, and conclusions drawn in this publication are those of the author(s), and do not reflect the opinions or policies of the Data Steward(s).
