# Supplementary material for: Investigation of treatment delay in a complex healthcare process using physician insurance claims data: an application to symptomatic carotid artery stenosis
Source: BMC Health Serv Res. 2024 Nov 29;24:1507. doi: 10.1186/s12913-024-11860-w (PMC11605873; doi:10.1186/s12913-024-11860-w)
Supplement: Supplementary file 1 — Supplementary Material 1. [34–37] [file 12913_2024_11860_MOESM1_ESM.docx]

Data supplement

# Supplementary tables

Supplementary Table 1. Contrasting structured and unstructured processes.

| Type of process | Structured | Unstructured |
| --- | --- | --- |
| Example | Booking CT scan | Diagnosing cause of shortness of breath |
| Design | Deliberate, top-down | Emergent, bottom-up |
| Objective | Clearly known from start | Initially vague, evolves through process |
| Orientation | Imperative – step-by-step | Declarative – goal-by-goal |
| Decisions | Pre-specified rules | Individualized judgment |
| Control | Tight via management | Loose via feedback |
| Variations | Few | Many |

Supplementary Table 2. Relevant diagnoses and associated ICD-9 codes.

| ICD-9 | Diagnosis |
| --- | --- |
| 341 | Other demyelinating diseases of central nervous system |
| 348 | Other conditions of brain |
| 349 | Other and unspecified disorders of the nervous system |
| 355 | Mononeuritis of lower limb |
| 360 | Disorders of the globe |
| 362 | Other retinal disorders |
| 368 | Visual disturbances |
| 369 | Blindness and low vision |
| 379 | Other disorders of eye |
| 431 | Intracerebral hemorrhage |
| 433 | Occlusion and stenosis of precerebral arteries |
| 434 | Occlusion of cerebral arteries |
| 435 | Transient cerebral ischemia |
| 436 | Acute but ill-defined cerebrovascular disease |
| 437 | Other and ill-defined cerebrovascular disease |
| 438 | Late effects of cerebrovascular disease |
| 440 | Atherosclerosis |
| 443 | Other peripheral vascular disease |
| 781 | Symptoms involving nervous and musculoskeletal systems |
| 784 | Symptoms involving head and neck |
| V72 | Examination of eyes and vision |

Supplementary Table 3. Imputed times-of-day used to place same-day events in consistent order.

| Activity | Imputed time |
| --- | --- |
| Outpatient service location | |
| gp | 01:00:00 |
| eye | 02:00:00 |
| neuro | 03:00:00 |
| surg | 04:00:00 |
| cus | 05:00:00 |
| cta | 06:00:00 |
| anes | 07:00:00 |
| im | 08:00:00 |
| cardio | 09:00:00 |
| stress | 10:45:00 |
| Inpatient service location | |
| gp | 12:00:00 |
| er | 12:00:00 |
| cta | 13:00:00 |
| neuro | 14:00:00 |
| eye | 15:00:00 |
| cus | 16:00:00 |
| surg | 17:00:00 |
| admit | 18:00:00 |
| anes | 19:00:00 |
| im | 20:00:00 |
| cardio | 21:00:00 |
| stress | 22:45:00 |
| cea | 23:00:00 |

Supplementary Table 4. Activity-specific waiting times calculated using the referral and directly-follows methods. Directly-follows times were included only if a time could also be calculated using the referral method.

| Activity | N | Calculated waiting time Median (Q1 – Q3) | | P-value* |
| --- | --- | --- | --- | --- |
|  |  | Referral method | Direct method |  |
| eye | 64 | 1 (0 – 4) | 1 (0 – 4) | 0.2 |
| neuro | 39 | 6 (4 – 11) | 6 (2 – 9) | 0.005 |
| cus | 48 | 3 (0 – 17) | 2 (0 – 14) | 0.4 |
| cta | 73 | 0 (0 – 2) | 0 (0 – 1) | 0.044 |
| eye_REV_ | 21 | 2 (0 – 12) | 2 (1 – 8) | 0.2 |
| neuro_REV_ | 37 | 1 (0 – 8) | 1 (0 – 5) | 0.029 |
| surgery | 64 | 4 (1 – 8) | 1 (0 – 6) | <0.001 |
| anes | 84 | 7 (1 – 12) | 3 (1 – 8) | <0.001 |
| cardio | 21 | 6 (1 – 13) | 1 (0 – 6) | <0.001 |
| stress | 23 | 6 (4.5 – 9) | 6 (3.5 – 7) | 0.045 |
| im | 14 | 7.5 (2.3 – 11.8) | 1 (0 – 1.8) | 0.008 |

*Paired t-test

Supplementary Table 5. Activity-specific waiting times stratified by referral source.

|  | Referring from (N; Median (Q1 - Q3) | | | | | p-value |
| --- | --- | --- | --- | --- | --- | --- |
| Activity | gp | eye | er | neuro | surgery |  |
| eye | N = 27;  1 (0 – 5) | N = 55;  1 (0 – 5) |  |  |  | 0.3* |
| neuro | N = 22;  7 (5 – 13) | N = 23;  8 (3 – 15) | N = 10;  1 (1 – 1) | N = 7;  1 (1 – 6) | N = 13;  0 (0 – 2) | <0.001† |
| cta |  |  |  | N = 42;  0 (0 – 1) | N = 24;  0 (0 – 3) | 0.4* |
| cus | N = 17;  14 (4 – 21) | N = 8;  18 (7 – 29) |  | N = 19;  0 (0 – 0) |  | <0.001† |
| surgery | N = 20; 8 (6 – 16) |  |  | N = 37;  1 (0 – 5) |  | <0.001* |
| cardio |  |  |  | N = 6;  2 (1 – 4) | N = 11;  7 (5 – 18) | 0.061* |

*Wilcoxon rank-sum test
†Kruskal-Wallis H test

Supplementary Table 6. Demographic and treatment characteristics for K-means clusters. Nominal variables as N (%), continuous variables as median (Q1 – Q3). P-values refer to Wilcoxon rank-sum test (continuous) or chi-square tests (categorical).

| Characteristic | 1, N = 44 | 2, N = 43 | p-value |
| --- | --- | --- | --- |
| Age | 73 (65, 78) | 77 (72, 82) | 0.019 |
| Female | 14 (32%) | 11 (26%) | 0.5 |
| In-metro residence | 38 (88%) | 36 (82%) | 0.3 |
| Symptom to surgery, days | 18 (11, 37) | 57 (33, 101) | <0.001 |
| Surgery done as unscheduled procedure | 23 (52%) | 7 (16%) | <0.001 |
| Referring physician for carotid imaging |  |  | <0.001 |
| Family | 0 (0%) | 21 (49%) |  |
| Neurology | 34 (77%) | 15 (35%) |  |
| Activity count | 9 (7 – 11) | 10 (8 – 13) | 0.011 |

Supplementary Table 7. Selected publications referencing process mining and quality improvement.

| Reference | Context | Problem statement | Change ideas identified by process mining |
| --- | --- | --- | --- |
| [34] | Patients receiving pre-hospital care for motor vehicle collision in Queensland, Australia from 2015-2017 | No clear problem statement | Automating data collection, store data using a unique patient identifier, automate determination of transport destination, use AI to support emergency call triage |
| [35] | Trauma team activations before and after implementation of an Advanced Trauma Life Support (ATLS) checklist | Non-adherence to ATLS protocol | None. Process mining was used to test the hypothesis that adherence to ATLS protocol improved after a checklist was implemented but was not used to generate a change idea for process improvement. |
| [36] | Patients receiving pre-hospital paramedic care in Iran in 2009 | Reduce deaths occurring during pre-hospital (ambulance) transport | Change idea categories, such as ‘consumption of drugs and equipment monitoring’ are listed. However, it is unclear how these ideas are connected to the process mining analysis. |
| [37] | Patients receiving care for breast cancer in either of the two largest public cancer hospitals in Mexico City between 2016-2017 | Breast cancer mortality is identified as a key outcome, but is not a target of an improvement project | Initial assessment in a private facility is correlated with shorter time to diagnostic confirmation of cancer. However, this finding isn’t linked to a process change idea. |

# Supplementary Figures

Supplementary Figure 1. Contrasting organization of process with flowcharts (e.g. Business Process Model and Notation) and case diagrams (e.g. Case Management Model and Notation). Flowcharts specify the precise selection and order of activities, whereas case diagrams identify available activities without necessarily specifying their sequence. Allowed behavior is generally more constrained with flowcharts compared to case diagrams. Flowcharts include standard annotations for time, revealing delay factors including activity queues and decisions, whereas these elements are absent from case diagram.

Supplementary Figure 2. Illustration of imaging review activity. Imaging reports are always returned to ordering physicians, who must decide on next actions. However, this decision might be made without an in-person visit and might therefore be structurally missing from claims data. If this activity is left missing, process mining algorithms will incorrectly attribute imaging activities as the cause of subsequent activities. In this Figure, a GP refers a patient for carotid CTA, for which a report is issued. The report is delivered to the GP (dotted arrow), who, upon reading it, immediately decides to refer the patient to a vascular surgeon. The GP notifies the patient of the finding and the plan by phone, which is not an insured service, and is therefore missing from claims data (light grey outline). If this imaging review activity is left missing, the CT angiogram might be incorrectly identified as the starting point of the surgical visit. Adding the missing activity eliminates this error.


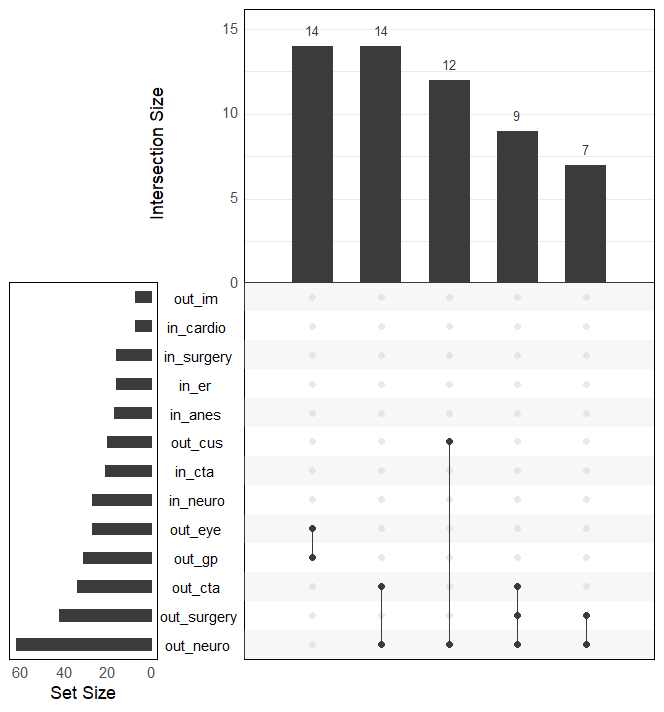


Supplementary Figure 3. UpSet plot of counts of same-day activity combinations. Individual activities and count of their same day occurrence appear to the left. Prefixes out and in refer to outpatient and inpatient service location. In the center, dotted lines illustrate sets of activities observed together. The number of observed sets is indicated by the top bar plot.


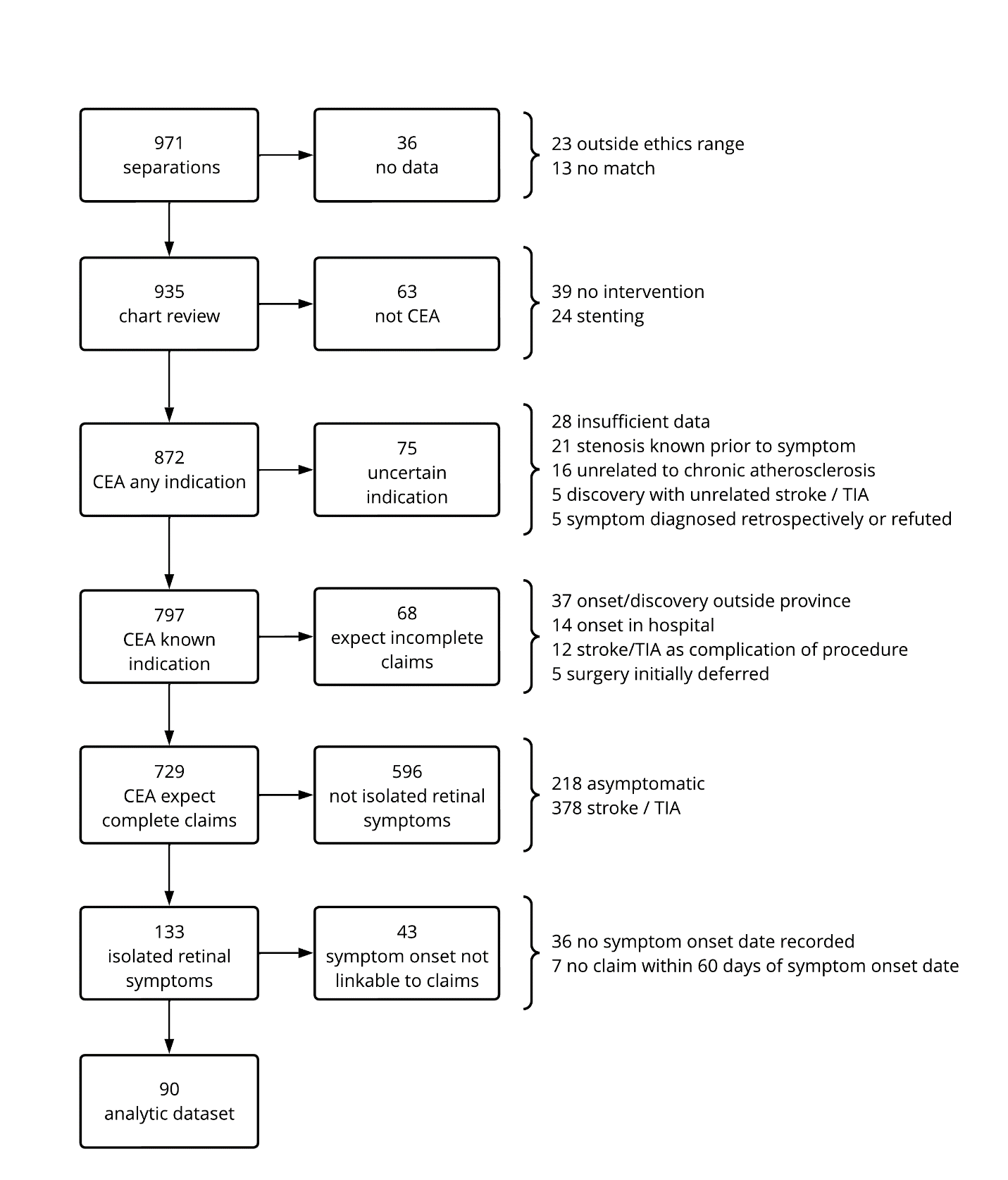


Supplementary Figure 4. Participant flow diagram.


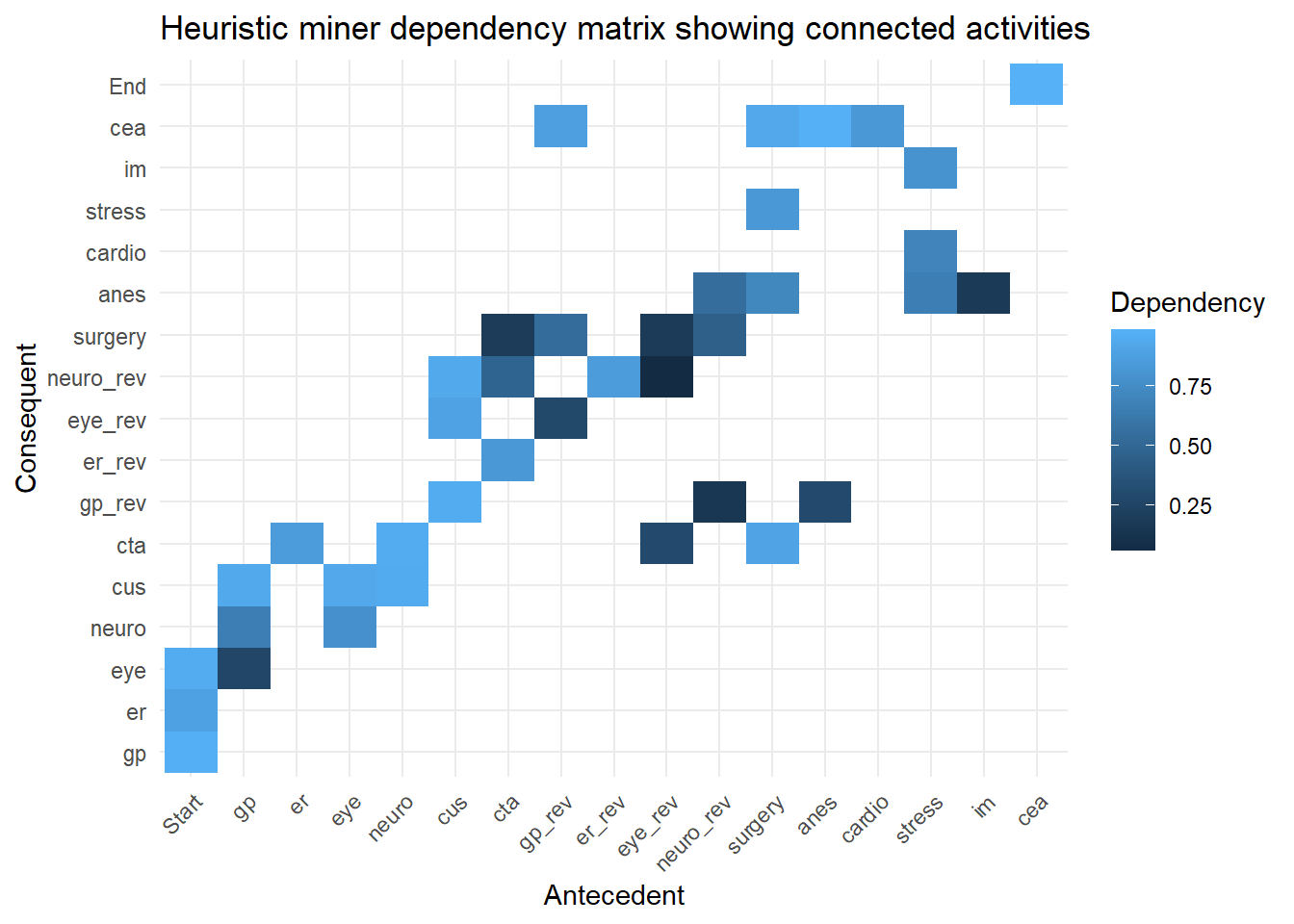


Supplementary Figure 5. Heuristic miner dependency plot for symptom-to-surgery process. Dependency is a measure of consistent directionality between two activities A and B. It can range from -1 (B always followed by A) to +1 (A always followed by B). Most of the identified dependencies align with the knowledge-model, but a few relations are suspect (e.g. gp_rev → cea).


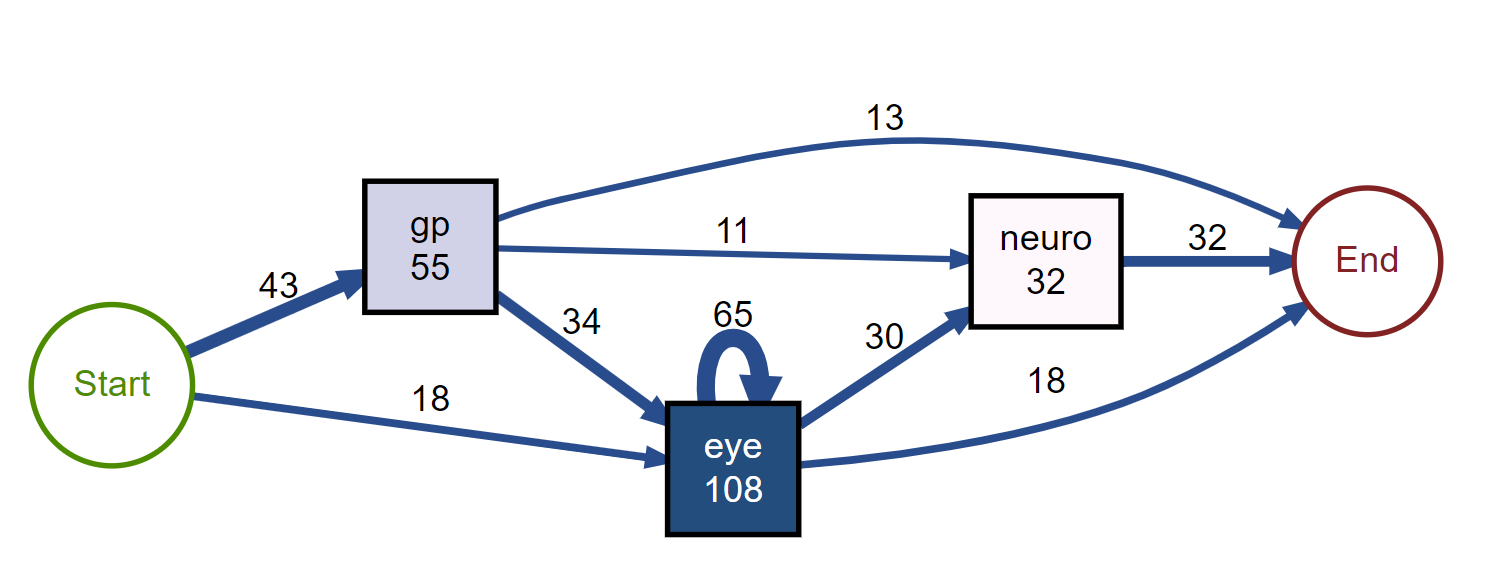


Supplementary Figure 6. Heuristic Miner frequency map of the symptom evaluation stage for patients in the primary-care cluster (N = 53 patients). The diagram identifies activities (boxes) and sequences (arrows), both with respective counts. Activity combinations lead to inconsistency between activity and sequence counts. For example, there are 32 neuro visits (box) but incoming arrows from gp and eyes sum to 41. The discrepancy (n = 9) relates to patients who had both a gp visit and and eye visit before their neuro visit.


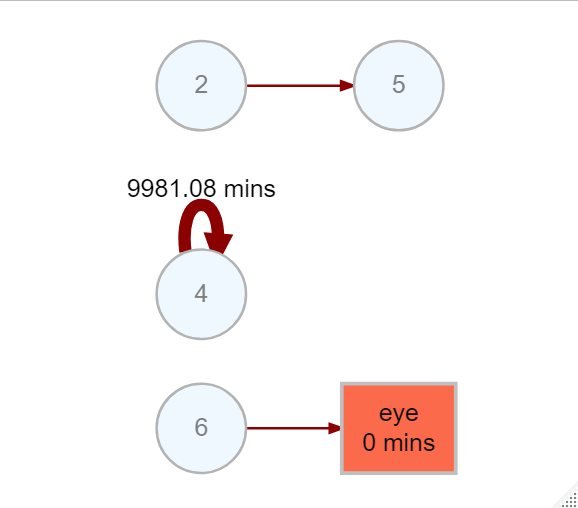


Supplementary Figure 7. Example of Heuristic Miner waiting time (performance) map generated for the symptom-stage activities of n = 25 randomly selected primary-care cluster patients. Maps would not generate for larger samples. The map is unintelligible.


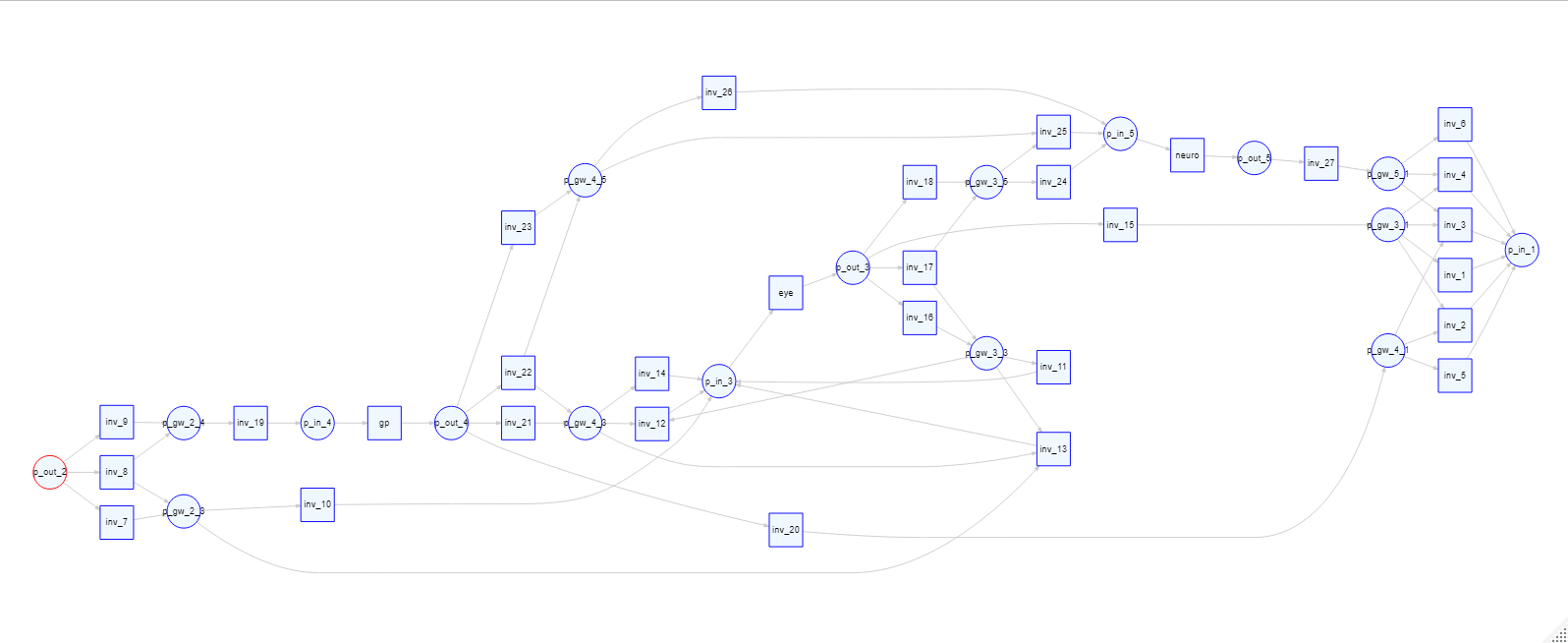


Supplementary Figure 8. Petrinet representation of Heuristic Miner process map of symptom-stage activities (gp, er, neuro, and eye) for n = 53 primary care cluster patients. The diagram contains 47 nodes (boxes and squares) and 71 flows (connecting arrows). We consider it uninterpretable.


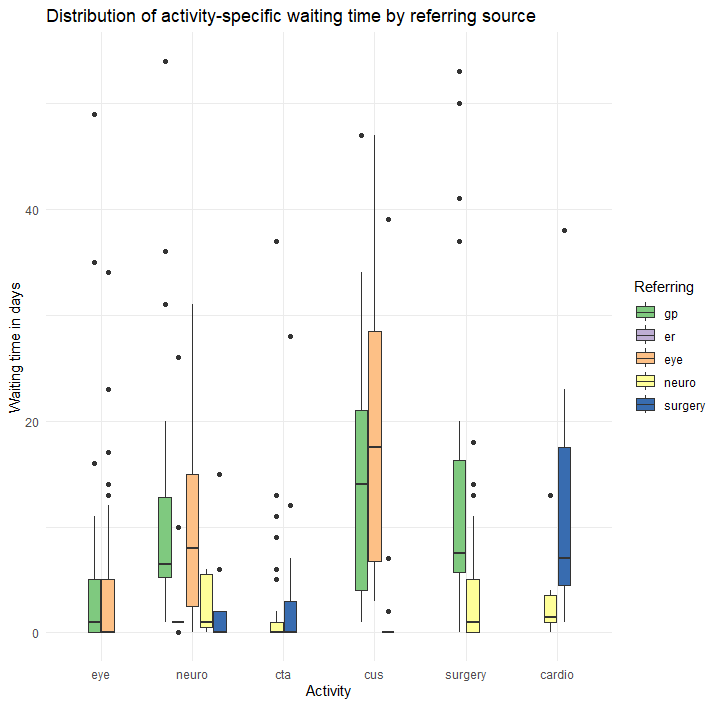


Supplementary Figure 9. Activity-specific waiting times calculated using referral-based method, stratified by referral source. Waiting times for activities are generally longer if referred by general practitioners or eye specialists.
